# A Case of Double Inlet Left Ventricle in a 63-Year-Old Female Patient

**DOI:** 10.7759/cureus.58978

**Published:** 2024-04-25

**Authors:** Alan E Benelli, Nicolas D Benelli, Ivan Buitrago

**Affiliations:** 1 Internal Medicine, St. George's University School of Medicine, St. George's, GRD; 2 Cardiology, Jackson Memorial Hospital, Miami, USA

**Keywords:** congestive heart failure, cardiac ct congenital heart diseases, single ventricle physiology, double inlet ventricle or single ventricle or univentricular heart, adult congenital heart disease

## Abstract

This case report describes the medical history and presentation of an elderly patient who was born with single ventricle physiology, an anomaly that is both unique and complex. Patients with single ventricle cardiac anomalies may be susceptible to life-threatening complications. However, advances in medical treatment and understanding have allowed for clinicians to develop surgical and medical interventions to treat patients with univentricular cardiac defects. This case is unique in the sense that the patient has been able to demonstrate remarkable adaptability to this condition and have a sustained life with little intervention. This report serves to explore the pathophysiology of this condition as well as highlight the human body’s astounding resilience to configure itself to abnormal conditions. The patient’s presentation will be discussed as well as diagnostics and management utilized by the care providers. Despite its rare occurrence, understanding the manifestations of this complex cardiac abnormality can allow future providers to improve the prognosis and outcomes of patients born with a single ventricle.

## Introduction

With the start of its embryogenesis during the third week of gestation, the heart eventually forms into a four-chambered pump that works in synchronization to receive and push blood throughout the human body. Oxygen-depleted blood returns to the right atrium before flowing to the right ventricle and finally being pumped into the lungs for oxygenation. Once oxygenated, the blood returns to the left atrium and ventricle, and conclusively gets pumped throughout the body’s many organs [1]. This coordinated movement of blood powered by the electrical conductivity of the heart is critical to ensure the organs are properly perfused. Compromise to the heart’s normal structure or function may lead to detrimental effects for an individual, warranting the need for medical intervention [1].

A normal functioning heart must undergo a particular embryogenic process to ensure proper anatomical position and compartmentalization. An intricate sequence of cell proliferation and tissue separation will allow for a single tube to give rise to a four-chambered heart. The interventricular septum consists of a membranous and muscular portion. Ventricular septation begins with the growth of muscular tissue from the floor of the primitive ventricle around the fifth week of development [2]. An interventricular foramen is made as the muscular septum grows towards the endocardial cushion. Bulbar ridges and the endocardial cushion give rise to the membranous portion of the septum. Ridges within the truncus arteriosus develop into the aorticopulmonary septum, which will progress caudally and fuse with the muscular septum. The fusion of the aorticopulmonary and muscular septums will result in the closure of the interventricular foramen. The final step involves the aorticopulmonary septum rotating 180 degrees to divide the truncus arteriosus into the aorta and pulmonary trunk, both rising from their corresponding ventricles. Proper facilitation of these embryological steps will result in a separate left and right ventricle, allowing for proper separation of oxygen-rich blood from oxygen-depleted blood [3].

In about one of every million individuals, an error in embryogenesis leads to the development of only a single heart ventricle [4]. There are a handful of cardiac defects that result in a single ventricle. The patient in this case was found to have a double inlet left ventricle (DILV). Possible explanations may include failure of the endocardial cushions or floor of the primitive ventricle to project growth of septum tissue. These errors in embryogenesis may occur more commonly during the sixth week of development [3]. This defect involves the mitral valve and the tricuspid valve both opening into the left ventricle, resulting in a hypoplastic right ventricle. Patients with DILV may also have other abnormalities such as transposition of the great vessels, pulmonary stenosis, and pulmonary atresia [5].

The absence of an interventricular septum consequently leads to oxygen-rich blood being mixed with oxygen-depleted blood. This mixture of blood is pumped to the rest of the body, which can lead to organs being sub-optimally oxygenated. Complications of a single ventricle include congestive heart failure, arrhythmias, thrombus formation, and lung with increased Qp/Qs as a result of pulmonary edema [4].

Treatment of a single ventricle cardiac defect will depend on the presentation and etiology of the particular defect. For patients with DILV, surgical procedures are often done at birth or during the first few weeks of life. One of these procedures includes the Glenn shunt, where the superior vena cava is anastomosed with the right pulmonary artery. This surgery may also improve the transition for an infant needing to undergo a Fontan procedure [6]. A common variation of this procedure is the third-generation Fontan, which involves disconnecting the inferior vena cava from the right atrium and connecting it to the pulmonary artery with a conduit. Once the conduit has been placed, a hole is made to establish a connection between the conduit and the right atrium.

Even with early surgical intervention, patients with DILV will need to optimize their health to avoid complications and further surgical measures. The prognosis for patients with DILV will be dependent on many factors. Given that these patients are at risk for later complications, primary care will play a crucial role in prolonging survival. These measures include ensuring that patients are screened for other co-morbidities such as hypertension, diabetes, and hyperlipidemia. Patients will need to have frequent appointments with a cardiologist and have studies done to assess the function and blood flow within their heart [7].

## Case presentation

Prior to the writing of this report, the patient's consent was properly discussed and obtained. This report involves a 63-year-old female patient with a history of secondary hemochromatosis, hypothyroidism, paroxysmal atrial fibrillation, and chronic heart failure (CHF) secondary to a DILV. The patient identified as a lifetime non-smoker and denied the use of illegal substances, alcohol, or consumption of caffeine. Past surgical and medical history significant for one previous miscarriage and one elective abortion. The patient was referred to the emergency room by her cardiologist for further evaluation of decompensated CHF. During evaluation, the patient reported an increase in swelling of her feet for approximately a week, inability to walk her usual 20-30 minutes per day, and shortness of breath even during rest. In further discussion, the patient reports having a baseline pulse oxygenation of 60-70% and being compliant with all her medications including diuretics and antiarrhythmics on a daily basis.

On physical examination, the patient was a thin short woman with moderate signs of respiratory distress, alongside notable findings including cyanosis of lips, tongue, fingertips, and toes. Respiration was labored with crackles heard bilaterally on auscultation, and edema was noted bilaterally in the lower extremities. Cardiac auscultation revealed S1 and S2 with S3 present. Vitals on admission showed a blood pressure of 123/75, respiratory rate of 22 breaths per minute, oral temperature of 36.4, and oxygen saturation of 57%. A clinical decision was made to start the patient on a venti-mask at a fraction of inspired oxygen (FIO2) of 40% and the patient was started on budesonide BID, encouraged incentive spirometry, and nebulizer to promote mucociliary clearance via cough reflex. As preventive measures, the patient was also started on pantoprazole and apixaban, and placed on nothing per os (NPO). As seen in Table 1, the patient's laboratory studies reflected a state of heart failure with significantly high levels of brain natriuretic peptide (BNP) and slightly elevated levels of troponin. A compensatory increase in hemoglobin was also noted in her labs as expected due to the patient's chronic state of hypoxemia and prior diagnosis of secondary hemochromatosis.

**Table 1:** The patient's laboratory values on admission AST: aspartate transferase; ALT: alanine transaminase; PCO2: partial pressure of carbon dioxide; PO2: partial pressure of oxygen; INR: international normalized ratio; APTT: activated partial thromboplastin time; HCO3: serum bicarbonate

Parameter	Patient value	Reference range
Glucose (mg/dL)	160 (High)	<125
Sodium (mmol/L)	136	136-146
Potassium (mmol/L)	4.8	3.5-5.0
Chloride (mmol/L)	93 (Low)	95-105
Serum osmolality (mOsm/kg)	275	275-295
Blood urea nitrogen (mg/dL)	31 (High)	7-18
Creatinine (mg/dL)	1.1	0.6-1.2
Calcium (mg/dL)	6.8	8.4-10.2
Total protein (g/dL)	5.8	6.0-7.8
Albumin (g/dL)	2.4	3.5-5.5
Total bilirubin (mg/dL)	1.4 (High)	0.1-1.0
AST (unit/L)	33	12-38
ALT (unit/L)	23	10-40
Alkaline phosphatase (unit/L)	190 (High)	25-100
pH	7.37	7.35-7.45
PCO2 (mm Hg)	58 (High)	33-45
PO2 (mm Hg)	31 (Low)	75-105
HCO3 (mmol/L)	33 (High)	22-28
Prothrombin time (seconds)	16.5 (High)	11-15
INR	1.3 (High)	<1.1
APTT (seconds)	33	25-40
Troponin (ng/mL)	0.058 (High)	<0.04
Brain natriuretic peptide (pg/mL)	10,200 (High)	<100
Red blood cell count	7.17 x 10^6 ^(High)	4.3-5.9 x 10^6^
Hemoglobin (g/dL)	18.6 (High)	12-16
Hematocrit (%)	64.3 (High)	36-46
Platelet count	112 x 10^3^ (Low)	115-400 x 10^3^

**Figure 1:**
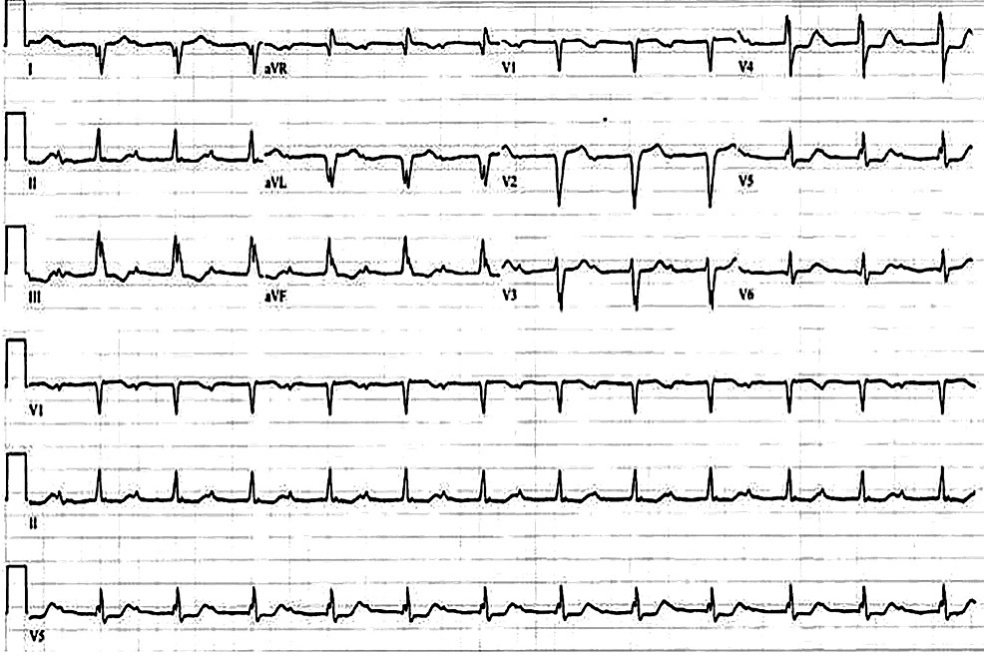
12-lead electrocardiogram of the patient upon admission

**Figure 2:**
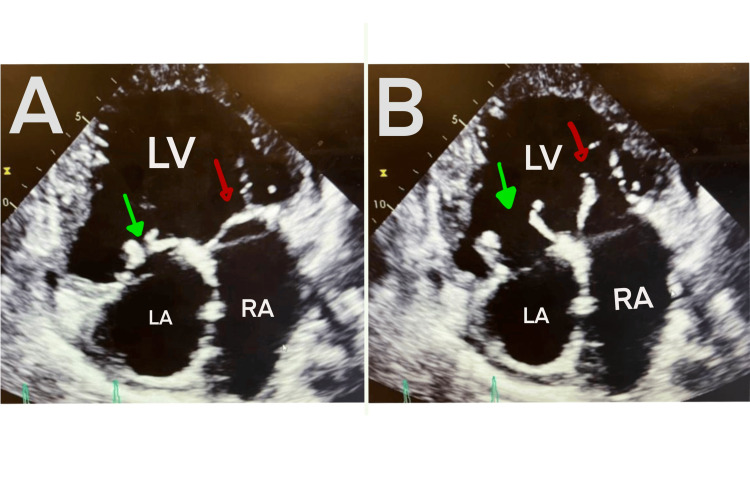
Apical 2D echocardiogram of the patient displaying a single ventricle Panel A: with valves closed; Panel B: with valves open; Red arrow: tricuspid valve; Green arrow: mitral valve; LA: left atrium; RA: right atrium; LV: left ventricle

Electrocardiogram (ECG) showed sinus rhythm with first-degree AV block and T wave inversions in leads III and aVF signifying potential inferior ischemia as shown in Figure 1. The ventricular rate was 74 bpm, PR interval 408 ms, QRS duration 132 ms, QT correction with Bazett formula 457 ms, and P-R-T axes 73-121-15. Significant tricuspid regurgitation and severe left atrium dilation were found on the echocardiogram alongside evidence of pulmonary hypertension as shown in Figure 2. Detailed parameters of the patient's echocardiogram as well as anatomical findings can be seen in Tables 2-3, respectively.

**Table 2:** Dimensions and volumes of the patient's heart as measured by echocardiogram

Parameter	Patient value	Normal range
Interventricular septum thickness at end-diastole (cm)	1.30 (High)	0.6-1.0
Left ventricular internal dimension at end-diastole (cm)	6.9 (High)	2.2-3.0
Left ventricular internal dimension at end-systole (cm)	5.6 (High)	2.5-4.0
Left ventricular posterior wall thickness at end-diastole (cm)	1.39 (High)	0.6-1.0
Left ventricular mass mass (g)	458 (High)	88-224
Left ventricular relative wall thickness	0.39	0.24-0.42
End diastolic volume (ml)	244.8 (High)	34-74
End systolic volume (ml)	151.4 (High)	21-61
Ejection fraction biplane (%)	57	52-72

**Table 3:** Valvular and vascular observations as stated on the echocardiogram transcript

Structure	Finding on echocardiogram
Mitral valve	Mild regurgitation
Aortic valve	No hemodynamically significant valvular aortic stenosis. Mild regurgitation present.
Tricuspid valve	Severe regurgitation is present with evidence of severe pulmonary hypertension.
Pulmonic valve	Normal in function and structure
Right atrium	Mildly dilated
Left atrium	Severely dilated
Great vessels	Aortic root is normal in size. Inferior vena cava is dilated without retrophasic variation.

The patient’s list of diagnoses was expanded to include DILV, CHF exacerbation, cyanosis, hypoxia, pulmonary hypertension, secondary hemochromatosis, hypothyroidism, and erythrocytosis due to cyanotic congenital heart disease. Within two hours of arrival to the emergency room, the patient's oxygen saturation increased to 76% via nasal cannula. Further intervention was followed by the addition of sacubitril/valsartan (24/26 mg p.o B.I.D), metolazone (5 mg p.o daily), amiodarone for underlying atrial fibrillation (100 mg p.o b.i.d), apixaban (2.5 mg p.o b.i.d), bumetanide (1mg IV b.i.d), dapagliflozin (10 mg daily), carvedilol (3.125 mg p.o b.i.d). Potassium-sparing diuretics were not used due to the patient's borderline potassium levels. Alongside medication, additional steps included a low salt diet and fluid restrictions to less than 1.5 liters per day with strict monitoring of fluid input and output. By the second day inpatient, oxygen saturation increased to 80% and the patient was maintained on a nasal cannula at 3 liters per minute. The patient’s edema and dyspnea improved slightly throughout her stay for the first four days with oxygen saturation maintained between 80% and 85%. Due to higher levels of thyroid-stimulating hormone (TSH) compared to lab values, six months earlier patient was started on low-dose levothyroxine at 25 mg. Bumetanide (1mg=4mL IV) was also added to help decrease the fluid overload and symptoms of congestion. Due to the patient's insignificant improvement, a decision was made on the fifth day to transfer the patient to an associated hospital for right-side heart catheterization and follow-up with a pulmonary hypertension specialist. On day 6, the internal care unit reported that the patient decompensated through the night with a decrease in oxygen saturation and an increase in carbon dioxide levels. The patient was placed on a bilevel positive airway pressure (BiPAP) to help stabilize and transfer to a different hospital for more definitive evaluation and treatment.

## Discussion

DILV is a complex congenital heart defect that is seen in less than 2% of all congenital heart abnormalities [8]. One of the key aspects in patients, such as the one presented in this study, is the change in both anatomy and physiology. Due to the lack of a ventricular septum, the location of the heart's conduction system such as the bundle of His becomes altered. In a study by Bharati and Lev, they explained how an accessory AV node was found on the right atrium which traveled through the right side of the pulmonary annulus to form the bundle of His. The bundle of His traveled through what they described as the septum separating the primary ventricle (left ventricle) and a small outpouch of tissue similar to the one previously seen in Figure 1 [8]. In patients such as the one in this study, such variation should be taken into consideration during interpretations of ECG. Possible findings on an ECG can include atypical QRS on leads V1 and V2 or possibly an Rs appearance in leads V5 and V6 [9]. Additionally, physicians should be mindful of the false septum and pulmonary annulus during interventions as lesions here could impact the conduction system of the heart [8]. This is an important aspect of the condition of the patient in this study since cardiac catheterization was planned for evaluation.

Pulmonary hypertension is another complication when evaluating the patient in this study. One possible explanation is an increase in pulmonary arterial pressure similar to Eisenmenger syndrome seen in patients with ventricular septal defects [10]. Such patients have chronic increased pressure in the pulmonary artery. Over time, elevated pressures lead to calcification and remodeling of pulmonary vasculature causing an increase in resistance [11]. These changes can lead to stress on the right side of the heart resulting in congestive heart failure and an increase in pressures, as seen in the patient’s right ventricular pressure of over 100 mm Hg. Such changes are common in patients with DILV, with approximately 66% of patients having signs and symptoms of stenosis of the pulmonary valve or outflow tract correlating to heart failure [12]. In a study of 146 patients with a single ventricle by Francisco Buendia-Fuentes, over 34% of patients were hospitalized for congestive heart failure [12]. These findings align with the presentation of the patient in this study. The accumulation of said factors plays a role in causing the congestive heart failure and associated symptoms seen in our patient which are reflected in the increase of brain natriuretic peptide, hypercapnia, hypoxemia, and dilation of inferior vena cava on echocardiogram.

Further considerations for our patient included possible treatments, complications, and long-term outcomes for the patient. Francisco Buendia-Fuentes reported that 7.5% of patients received a heart transplant while nearly 6.2% and 2.7% received a pacemaker or implantable cardioverter-defibrillator, respectively [13]. Other possible treatments for patients with DILV include ventricular septation and Fontan procedure. Such procedures, however, carry risks, and certain criteria are typically required for the conduction of surgery and expected positive outcomes. For ventricular septation, two unobstructed AV valves as well as an unobstructed aortic annulus are needed for proper placement of artificial septum [14]. The Fontan procedure is typically done in a three-step sequence with the goal of attaching the vena cava to the pulmonary artery to allow the system to be maintained by a single ventricle [8]. Both procedures, however, are typically reserved for young patients and are typically utilized in early life to avoid complications that may develop later on. In a patient such as the one seen in this case, both the patient’s age and progressed condition would likely make her a poor candidate for such extensive and invasive procedures. Other palliative interventions may include pulmonary artery banding, atrial septectomy, and the Blalock-Taussig shunt. A last resort option for patients would be a heart transplant [8]. Complications have also been reported with such procedures such as arrhythmias, obstruction, thrombus formation, elevated pulmonary vascular resistance, and formation of vascular connections between systemic and pulmonary pathways [15]. 3D printing is currently being investigated for its use in patients with congenital cardiac abnormalities to help aid in surgical decision-making. Pharmacological treatment, typically used as an adjunct to surgery, can be an alternative for patients who do not qualify for surgery. Medications such as anticoagulants, angiotensin-converting enzyme inhibitors, inotropic agents, and diuretics may help reduce both symptoms and slow the progression of heart failure typically seen in these patients. As seen in older patients such as in this study, however, pharmacological interventions need to be routinely evaluated to minimize acute episodes of decompensation.

In patients with DILV, the long-term prognosis is dependent on early intervention and the extent of pulmonary obstruction [8]. In the study by Francisco Buendia-Fuentes, an 86% survival rate was reported at a five-year follow-up with a 74% rate observed after 10 years [12]. Astonishingly, the patient in this study has been able to maintain an active and relatively healthy life into her 60s on pharmacological treatment alone without any surgical intervention. The patient has done adequate exercise throughout her life and has tried to maintain a well-balanced diet to reduce the risk of developing co-morbidities such as diabetes or atherosclerosis that may exacerbate her underlying heart defect. Another important factor to consider for this patient is the patency of the surgical interventions that were done at the beginning of life to treat her univentricular morphology. David Gregg reported a similar male patient in his 50s who had DILV yet maintained stable vital signs and reported an oxygen saturation of 80% [16]. Such cases may reflect that patients who are adequately managed pharmacologically to reduce vascular remodeling may indeed have relatively healthy lives and favorable long-term survival. Furthermore, pregnancy should be another consideration in female patients. In a report by Yumi Shiina, a woman with DILV who underwent a ventricular septation procedure had two successful term deliveries at the ages of 29 and 33. Throughout her pregnancies, the patient experienced palpitations but no major cardiovascular complications were reported [17]. Although the patient in our study never had any children, such cases as described by Yumi Shiina may show that patients with DILV treated by surgical intervention may indeed have healthy full-term pregnancies.

## Conclusions

This case report demonstrates the physiological adaptability of an elderly patient with a DILV. The longevity observed in this patient can offer insight into how congenital heart disease, particularly univentricular types, can progress in aging patients. Future implications may include ongoing monitoring of patients with congenital heart anomalies to further understand how treatment for these individuals can be optimized. Further research may also explore gene therapy, cell therapy, and tissue printing to treat patients with univentricular physiology. Despite this condition’s relatively rare occurrence, further literature and collaboration between cardiologists and other researchers will strengthen our clinical understanding of abnormal heart development and its treatment.
